# Duodenal Lymphomas: Comprehensive Evaluation of Endoscopic Features and Clinical Outcomes in a Tertiary Center

**DOI:** 10.3390/diagnostics16081173

**Published:** 2026-04-15

**Authors:** Zhiyu Yan, Yuheng Zhang, Congwei Jia, Yan Zhang, Shengyu Zhang, Aiming Yang

**Affiliations:** 1 Department of Gastroenterology, Peking Union Medical College Hospital, Chinese Academy of Medical Sciences & Peking Union Medical College, Beijing 100730, China; nirvanayn@outlook.com (Z.Y.); yuheng_z@126.com (Y.Z.); 2 Department of Medicine, Peking Union Medical College Hospital, Chinese Academy of Medical Sciences & Peking Union Medical College, Beijing 100730, China; 3 Eight-Year Medical Doctor Program, Peking Union Medical College, Chinese Academy of Medical Sciences & Peking Union Medical College, Beijing 100730, China; 4 Department of Pathology, Peking Union Medical College Hospital, Chinese Academy of Medical Sciences & Peking Union Medical College, Beijing 100730, China; david_jia0814@163.com; 5 Department of Hematology, Peking Union Medical College Hospital, Chinese Academy of Medical Sciences & Peking Union Medical College, Beijing 100730, China; zhangyan10659@pumch.cn

**Keywords:** duodenal lymphomas, endoscopy, prognostic factors, survival analysis

## Abstract

**Background/Objectives**: Duodenal lymphomas (DLs) are a rare subset of gastrointestinal lymphomas with incompletely characterized clinicopathological features due to low incidence and diagnostic challenges. This study assessed DL survival outcomes, characterized clinical/endoscopic features, and identified prognostic factors. **Methods**: This was a retrospective observational study of patients undergoing endoscopic examinations between 1 November 2002 and 1 November 2022 at Peking Union Medical College Hospital, with a subsequent histopathological diagnosis of DL. The primary outcome was overall survival (OS), and Cox proportional hazards modeling was used for survival analyses. **Results**: Sixteen patients (32%) had indolent B-cell lymphoma, 20 (40%) had aggressive B-cell lymphoma, and 14 (28%) had T-cell lymphoma. Diarrhea and weight loss were more common in patients with T-cell lymphoma. The most common endoscopic appearance was mucosal granularity, and 40% of patients had mass lesions. The median OS was 24.1 (95% CI: 13.3–117) months, with 1- and 5-year survival rates of 68.0% (95% CI: 56.2–82.2%) and 33.8% (95% CI: 22.3–51.44%), respectively. In multivariable analysis, a granular appearance (HR: 0.33, 95% CI: 0.11–0.99, and *p* = 0.049) and taking chemotherapy (HR: 0.22, 95% CI: 0.07–0.69, and *p* = 0.01) were associated with better OS, while T-cell lymphoma (HR: 9.19, 95% CI: 2.12–32.83, and *p* = 0.003) and stage IV lymphoma (HR: 12.76, 95% CI: 1.70–95.66, and *p* = 0.013) were associated with worse OS. **Conclusions**: This first integrated study provides new information on the clinical, endoscopic, and prognostic features of DL. While no specific clinical or endoscopic feature is diagnostic of DL, DL must remain in the differential diagnosis of any patient presenting with nonspecific gastrointestinal symptoms.

## 1. Introduction

Lymphomas are uncommon in the gastrointestinal (GI) tract, accounting for only 1–4% of all GI malignancies; however, the GI tract is the most common site for non-Hodgkin lymphoma (NHL), accounting for 30–40% of all extranodal lymphomas and 10–15% of NHLs [1]. The stomach is the most common site of GI lymphoma (~60%), but lesions can occur anywhere along the GI tract. Duodenal lymphomas (DLs) represent a heterogeneous subset of gastrointestinal lymphomas characterized by duodenal involvement at diagnosis. They have a prevalence ratio of 1:3:10 compared with other small intestinal and gastric lymphomas, respectively [2,3,4,5].

Despite the increasing incidence of GI lymphomas, DL remains an understudied entity. Most of the existing literature is confined to sporadic case reports [6,7,8,9,10] or broad database studies [11] that lack endoscopic details. The diagnostic challenge is exacerbated by nonspecific clinical presentations and the absence of definitive circulating biomarkers. Notably, no specific endoscopic feature has been identified as a prognostic biomarker for DL to date, leaving clinicians without visual metrics for real-time risk stratification during endoscopy. The clinicopathological landscape of DL exhibits significant geographical and ethnic variations. For instance, enteropathy-associated T-cell lymphomas (EATLs) and their monomorphic epitheliotropic counterparts (MEITLs) show distinct prevalence and behaviors in Asian populations compared with Western cohorts [12,13,14]. There is a critical research gap in integrating longitudinal endoscopic findings with histopathological subtypes and long-term survival outcomes to establish robust prognostic models [15].

Given these knowledge gaps, we performed a comprehensive, integrated analysis of DLs at a leading tertiary center in China over twenty years. This study aimed to (i) assess survival outcomes in this rare population; (ii) identify diagnostic clinical or endoscopic features that might suggest a diagnosis of DL; and (iii) determine prognostic factors to guide clinical decision making.

The key contributions of this work include the following:

- Comprehensive multimodal dataset: We present one of the largest single-center cohorts (*N* = 50) integrating clinical, endoscopic, and pathologic findings over two decades.
- Novel endoscopic prognosticator: We identify “mucosal granularity” as the first reported significant independent predictor of favorable overall survival in DL, providing a practical visual metric for immediate risk stratification during endoscopy.
- Subtype-specific survival analysis: We provide detailed survival data for aggressive T-cell vs. B-cell DLs, highlighting the unique challenges of managing MEITL in Asian patients.
- The remainder of this manuscript is structured as follows: Section 2 delineates the patient selection criteria, rigorous endoscopic/pathological evaluation protocols (including inter-observer agreement validation), and the statistical framework for survival analysis with Bootstrap resampling. Section 3 details integrated clinicopathological, endoscopic, and survival findings, with a focus on subtype-specific and Asian population-specific patterns. Section 4 provides an analytical comparison of our results with global literature, critically discusses study limitations, and outlines concrete future research directions. Finally, Section 5 summarizes the clinical implications and methodological contributions of this work for DL diagnosis and management.

## 2. Materials and Methods

### 2.1. Patients, Study Design, and Ethical Approval

This was a retrospective observational study of patients who underwent endoscopic examinations between 1 November 2002 and 1 November 2022 at the Department of Gastroenterology, Peking Union Medical College Hospital. This study reported the following STROBE reporting guidelines [16]. The inclusion criteria were adults aged 18 years or older who were subsequently diagnosed with DLs after histopathological examination of tissue biopsies. The exclusion criteria were as follows: (1) age < 18 years; (2) previous peripheral lymphadenopathy or any lymphoma-related systemic involvement; (3) past or concurrent complications with any other cancer; (4) having previously received any treatments for DL; and (5) lack of accessible or interpretable reports about endoscopic examinations and corresponding pathology. Demographic, clinical, endoscopic, and pathological findings were recorded.

### 2.2. Endoscopic and Histopathological Examinations

All enrolled patients underwent endoscopic examinations via white-light endoscopy (WLE) with a GIF-H260 or GIF-Q260 (Olympus, Tokyo, Japan), while some underwent chromoendoscopy (CE) and magnifying endoscopy with narrow-band imaging (ME-NBI). Only WLE results were utilized for analysis because of the consistency of the examination. Endoscopic photographs and characteristics of the targeted lesions were recorded and extracted before biopsy.

**Figure 1:**
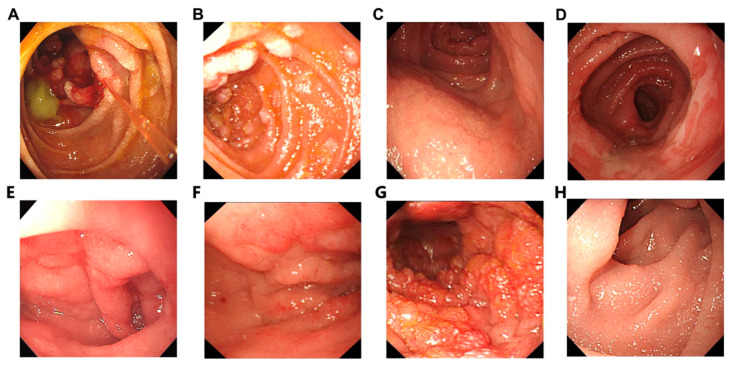
Representative endoscopic findings of duodenal lymphomas. (**A**) Ulcerative lesions. (**B**) Polypoid lesions. (**C**) Submucosal tumor-like lesions. (**D**) Diffuse-type lesions. (**E**) Enlarged fold type. (**F**) Erosion. (**G**) Granularity. (**H**) Villous blunting with lymphangiectasia.

This retrospective review of endoscopic images was conducted by two senior gastroenterologists, each with over 10 years of clinical experience in gastrointestinal endoscopy. All endoscopic photographs were extracted from the Medcon Medical Image Management System (Medcon, Beijing, China) and re-evaluated independently. Before the formal analysis, five representative cases were selected for a preliminary consistency evaluation. Eight primary endoscopic features were independently assessed by the endoscopists. The inter-observer agreement was calculated using Cohen’s kappa (κ) statistics, yielding a coefficient of 0.89 (Supplementary Table S1), which indicates a strong inter-observer agreement. For discrepant cases in the full cohort, a third senior gastroenterologist (≥15 years of GI endoscopy experience) was involved to reach a final consensus via joint review. The following WLE endoscopic characteristics were recorded: (1) the location, size, origin, and involvement of the GI tract; (2) the macroscopic morphology [tumor-forming type (including tumors with ulceration, polypoid lesions, and submucosal tumor-like lesions), diffuse type (including diffuse erosions and multiple ulcers), and enlarged fold type]; and (3) the surface configurations and appearances, including erosion, ulceration, granularity, villus blunting, and lymphangiectasia (Figure 1). In cases of disagreement, the three endoscopists discussed the case to reach a consensus, and the final endoscopic diagnosis was determined via agreement between at least two endoscopists.

**Figure 2:**
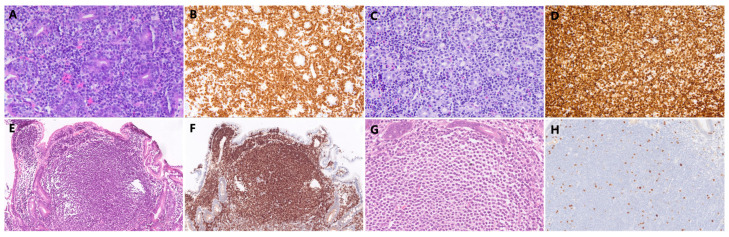
Representative histopathological sections of duodenal lymphomas. (**A**,**B**) Enteropathy-associated T-cell lymphoma: (**A**) infiltration of atypical small-to-medium-sized lymphocytes with irregular nuclei (HE, 400×); (**B**) strong and diffuse cytoplasmic expression of CD3 (400×). (**C**,**D**) Diffuse large B-cell lymphoma (DLBCL): (**C**) pleomorphic large tumor cells with prominent nucleoli invading the mucosal lamina propria and destroying glandular structures (HE, 400×); (**D**) extremely high Ki-67 proliferation index (>90%) indicating aggressive growth (400×). (**E**,**F**) Follicular lymphoma: (**E**) neoplastic follicles composed of centrocytes organized in a characteristic follicular pattern (HE, 100×); (**F**) strong BCL-2 positivity within the follicles, distinguishing them from reactive hyperplasia (100×). (**G**,**H**) Extranodal marginal zone lymphoma of mucosa-associated lymphoid tissue (MALT lymphoma): (**G**) neoplastic cells with abundant pale-staining cytoplasm and characteristic lymphoepithelial lesions (HE, 400×); (**H**) low Ki-67 proliferation index consistent with indolent B-cell lymphoma (400×).

DLs were classified according to the fifth edition of the World Health Organization (WHO) Classification of Hematolymphoid Tumors: Lymphoid Neoplasms [17]. Histopathological sections were independently re-evaluated by two experienced gastrointestinal pathologists, each with ≥10 years of experience in hematolymphoid tumor diagnosis, and discrepant diagnoses were resolved by a third senior hematopathologist (Figure 2).

### 2.3. Outcome

The primary outcome was overall survival (OS), which was measured from the date of pathological diagnosis to the date of last follow-up or death.

### 2.4. Statistical Analysis

Quantitative variables are reported as medians (interquartile ranges (IQRs)), and categorical variables are reported as numbers (percentages). Categorical variables were compared with Pearson’s chi-square test or Fisher’s exact test, as appropriate. Survival curves were estimated using the Kaplan–Meier approach, and groups were compared with the log-rank test. Univariable and multivariable survival analyses were performed using Cox proportional hazards models. A *p*-value of <0.25 in univariable analysis was used as a cutoff point for inclusion in multivariable models, since lower *p*-value thresholds, such as 0.05, would risk excluding clinically relevant variables and reducing the statistical power of the multivariable model [18,19]. To ensure the validity of these models, the proportional hazards (PH) assumption was strictly verified for all covariates using the Schoenfeld residuals test, which confirmed that the assumption was not violated for any of the covariates included. Model performance was evaluated using Harrell’s concordance index (C-index) to assess predictive accuracy. Furthermore, to address the constraints of the limited sample size (*N* = 50) and ensure the stability of our findings, a Bootstrap resampling method (with 1000 iterations) was employed for internal validation of the prognostic model, providing robust estimates of hazard ratios (HRs) and their 95% confidence intervals (CIs). Statistical significance was defined as a two-tailed *p*-value of <0.05. All statistical analyses were performed with Jamovi (version 2.6, The Jamovi project, Sydney, Australia) using the ClinicoPath (version v0.0.1.0004) and jsurvival (version 0.0.1) packages.

## 3. Results

### 3.1. Clinicopathological Characteristics and Clinical Presentation

The clinical and pathological characteristics of the study population are shown in Table 1. Forty-four percent (22/50) of the cohort were female, with a median (IQR) age of 56 (46, 70) years. Thirty-six patients (72%) had B-cell lymphomas, including aggressive subtypes (18 were diffuse large B-cell lymphoma (DLBCL), and two were mantle-cell lymphoma (MCL) [*N* = 2]) and indolent subtypes (follicular lymphoma and mucosa-associated lymphoid tissue (MALT) lymphoma, eight cases each). The remaining cases (14/50, 28%) were T-cell lymphomas, among which eight cases (57.1%, 8/14) were unclassified, four were enteropathy-type T-cell lymphomas, and one each was a peripheral T-cell or T-cell lymphoblastic lymphoma. A total of 35/50 (70%) patients were Ann Arbor stage IV, 5/50 (10%) were Ann Arbor stage III, and 10/50 (20%) were Ann Arbor stage II.

There was a wide range of clinical presentations, mainly gastrointestinal symptoms (Table 1). Fifteen patients (30%) were *H. pylori*-positive. Two-thirds reported more than 5 kg of weight loss. Half of the patients presented with abdominal pain, and approximately two-fifths to one-third presented with fever, abdominal distension, diarrhea, poor appetite, vomiting, and melena/hematochezia. Both diarrhea and weight loss were significantly more common in patients with T-cell lymphoma, occurring in 64.3% vs. 16.7% and 92.9% vs. 55.6% of patients with T- and B-cell lymphoma, respectively (chi-square test; *p* < 0.001 and *p* = 0.012, respectively).

### 3.2. Endoscopic Findings in Patients with Duodenal Lymphoma

The endoscopic characteristics of the patients with DL are shown in Table 2. The majority of lesions (72%) were observed in the descending duodenum with or without involvement of the duodenal bulb. The stomach, esophagus, and jejunum/ileum were also involved in 22%, 2%, and 4% of patients, respectively. The horizontal segment was not commonly involved (10% of cases).

The most common appearance was granularity (48% of cases; Figure 1G), and masses formed in 40% of cases; lesions were polypoid in only 10% of cases. Villous shortening, shallow folds, infiltrative changes, lymphangiectasia, and mucosal erosions were present in approximately one-fourth to one-third of patients. Lesions were ulcerated and multiple in 42% and 28% of patients, respectively.

The only endoscopic feature associated with lymphoma type was villous shortening, which was present in 57.1% of T-cell lymphomas but only 8.3% of B-cell lymphomas (chi-square test; *p* < 0.001).

### 3.3. Associations Between Clinical, Pathological, and Endoscopic Features and Overall Survival

The median (IQR) follow-up period was 20 (3–38) months. Thirty-two (64%) patients diagnosed with DLs died of their disease. The median OS for the entire cohort was 24.1 (13.3–117) months, with 1-year, 3-year, and 5-year survival rates of 68.0% (95% CI: 56.2–82.2%), 39.2% (95% CI: 27.3–56.4%), and 33.8% (95% CI: 22.3–51.44%), respectively. The majority of patients received rituximab, cyclophosphamide, doxorubicin, vincristine, and prednisone (R-CHOP)-based chemotherapy (31/50, 62%), of whom two T-cell lymphoma patients received gemcitabine, dexamethasone, cisplatin, and L-asparaginase (GDPL)-based chemotherapy, and three (6%) underwent surgery for acute intestinal obstruction (Table 1). There was no significant association between chemotherapy administration and lymphoma type (chi-square test; *p* = 0.623), but patients with a higher Ann Arbor stage were significantly more likely to receive chemotherapy (chi-square test; *p* = 0.018; Supplementary Table S2).

**Figure 3:**
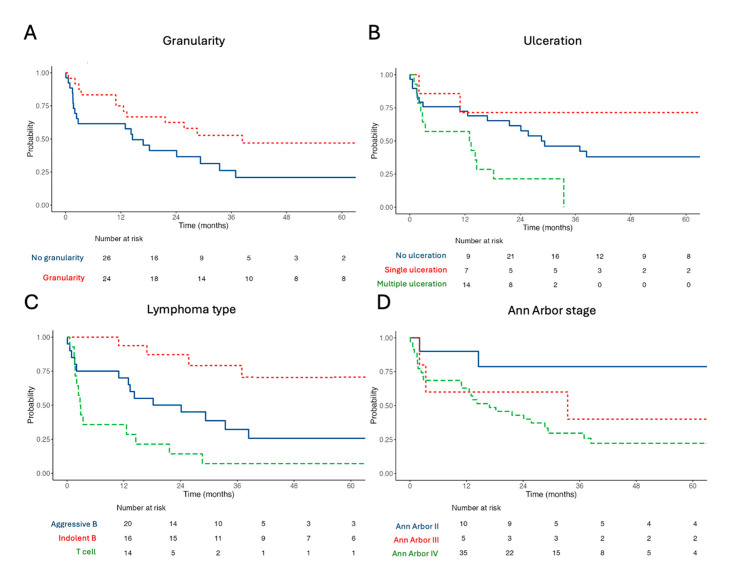
Significant univariable associations between the presence of granularity at endoscopy (**A**), multiple ulcerations (**B**), lymphoma major type (**C**), and Ann Arbor stage (**D**) and OS in patients with primary duodenal lymphoma.

In univariable analyses of associations between clinical, histopathological, and endoscopic findings and OS (Supplementary Tables S3–S5), only granularity (HR: 0.45, 95% CI: 0.22–0.92, and *p* = 0.029; Figure 3A), multiple ulcers (vs. no ulceration; HR: 2.60, 95% CI: 1.18–5.71, and *p* = 0.018; Figure 3B) at endoscopy, the lymphoma type (vs. aggressive B-cell lymphoma; indolent B-cell lymphoma HR: 0.23, 95% CI: 0.07–0.68, and *p* = 0.008; T-cell lymphoma HR: 2.27, 95% CI: 1.05–4.90, and *p* = 0.037; Figure 3C), and Ann Arbor stage IV (HR: 3.49, 95% CI: 1.05–11.57, and *p* = 0.041; Figure 3D) were associated with OS. There were no significant differences in survival outcomes for the different T-cell lymphoma subtypes (Supplementary Figure S1), but survival analysis confirmed the favorable prognosis of follicular lymphoma over B-cell lymphomas (log-rank *p* = 0.033; Supplementary Figure S2). There was a trend toward a favorable prognosis in patients with aggressive B-cell lymphomas taking chemotherapy (log-rank *p* = 0.16; Supplementary Figure S3).

**Figure 4:**
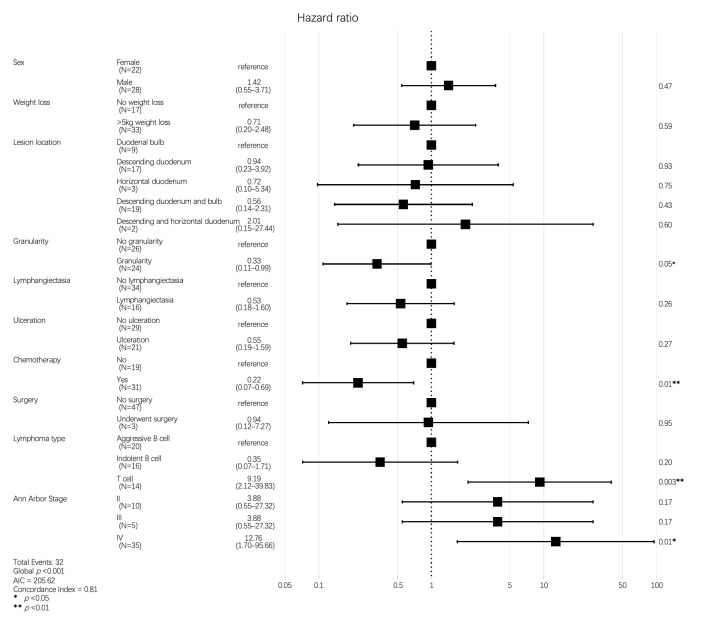
Hazard regression plot of variables included in the multivariable analysis of associations with OS.

Finally, to establish independent predictors of OS in patients with DLs, we constructed a multivariable model that included all variables with a *p*-value of <0.25 in the univariable analysis (Table 3). We also included chemotherapy, as it is clinically important to establish its benefit in terms of risk characteristics. In the multivariable analysis, a granular appearance at endoscopy (HR: 0.33, 95% CI: 0.11–0.99, and *p* = 0.049) and receiving chemotherapy (HR: 0.22, 95% CI: 0.07–0.69, and *p* = 0.010) were associated with a reduced risk of death from DLs, whereas having T-cell lymphoma (HR: 9.19, 95% CI: 2.12–39.83, and *p* = 0.003) and stage IV lymphoma (HR: 12.76, 1.70–95.66, and *p* = 0.013) were both associated with an increased risk of death (Figure 4 and Table 3). The multivariable model demonstrated good predictive performance, achieving a Harrell’s C-index of 0.78 (95% CI: 0.71–0.85). The stability of the hazard ratios for granularity and chemotherapy was further confirmed through 1000-iteration bootstrap internal validation, with a median HR of 0.32 (95% CI: 0.10–0.98) for granularity and a median HR of 0.21 (95% CI: 0.07–0.68) for chemotherapy, consistent with the original model results.

## 4. Discussion

All gastrointestinal lymphomas are uncommon, but duodenal lymphoma (DL) is particularly rare. The lack of primary data on this disease makes its diagnosis and management especially challenging. Here, we report a comprehensive integrated analysis of DL to explore its features to help with diagnosis and risk assessment. In our cohort of fifty patients, DL was clinically, endoscopically, and pathologically heterogeneous, presenting with a range of systemic and gastrointestinal symptoms, especially weight loss and abdominal pain. Similarly, the endoscopic features were variable, although a granular appearance was observed in approximately half of the patients. Our long period of clinical follow-up allowed us to establish that a granular appearance at endoscopy and chemotherapy were significantly associated with a reduced risk of death from the disease, whereas factors intrinsic to lymphoma, such as T-cell lymphoma classification and higher stage IV lymphoma, were both associated with a significantly increased risk of death from the disease in multivariable analysis.

DL is rare, so the majority of published reports are individual cases [11], and survival data are scarce. In the largest analysis of the clinical features of DLs, Zheng et al. [11] queried the Surveillance, Epidemiology, and End Results (SEER) database and identified 1060 cases over an 18-year period until 2015. Although the demographics of our study population were similar to those of the SEER cohort (56% male vs. 57% male in SEER; a median age of 56 years vs. a mean age of 61 years in SEER) and the known demographic profile of GI lymphomas [20], our cohort was enriched for T-cell (28% vs. only 3% in SEER) and higher-stage lymphomas (0% Ann Arbor stage I vs. 53% in SEER). Similarly, other studies have shown that T-cell lymphomas are much less common than B-cell lymphomas in the duodenum (<10% of only 21 cases in [21]). This atypical distribution reflects our status as a tertiary referral center and highlights a significant geographical disparity. Our analysis, nevertheless, provides new data on survival outcomes for patients with DLs. In the undifferentiated cohort, the observed median OS was 24.1 (13.3–117) months, with 1-year and 5-year survival rates of 68.0% (95% CI: 56.2–82.2%) and 33.8% (95% CI: 22.3–51.44%), respectively. While these outcomes are difficult to compare directly, the 10-year survival rate in the SEER analysis was 21.2%. The significantly worse outcomes for patients with T-cell lymphoma reflect the known intrinsic aggressive behavior of GI T-cell lymphomas, especially the enteropathy-associated type (EATL) [22], with most studies reporting a median OS of <10 months for EATL [12,23]. EATL has classically been regarded as a disease of northern Europe because of its association with celiac disease, which is uncommon in Asians [24]. However, monomorphic epitheliotropic intestinal T-cell lymphoma (MEITL, formally known as type II EATL), a monomorphic small-sized lymphoma with a distinct immunophenotype that is not associated with celiac disease, is more common in Asians [12]. Moreover, one study of EATL in a Chinese population revealed that the disease was exclusively MEITL [13]. In that cohort, 14 of 16 patients died of progressive disease, usually within one year [13]. Given the very poor survival outcome for patients with T-cell lymphoma in our cohort, clinicians and pathologists should be aware of this distinct subtype in the Asian population, as it has exceptionally poor outcomes, resistance to conventional therapy, and unique molecular features (histone methylation and activated JAK/STAT signaling) that might be useful for future individualized, targeted therapy [25]. Given that 8/14 patients in our cohort were not subtyped during routine histopathological analysis and given their abysmal survival outcomes (median survival: 2.1 (95% CI: 1.58-not reached) months), further exploration of whether any of these patients represented MEITL would be interesting. Unlike Western populations, where DL is dominated by B-cell subtypes, the Asian landscape is uniquely burdened by MEITL, which exhibits a more aggressive clinical course and poorer response to conventional CHOP-like regimens. Our finding of a 2.1-month median survival for unclassified T-cell cases underscores the urgent need for early molecular subtyping in Asian patients to implement targeted therapies, such as JAK/STAT inhibitors, rather than relying on standardized GI lymphoma protocols.

Our analysis allowed us to confirm the excellent survival outcomes expected for patients with follicular lymphoma arising in the duodenum, with only one of eight patients dying of their disease after 37 months [26]. Primary gastrointestinal follicular lymphoma [26], when arising in the duodenum, is recognized in the WHO classification as a special variant (duodenal-type follicular lymphoma) [17]. Consistent with the known characteristics of these lymphomas, five of eight patients presented with abdominal pain, and only three of eight patients received chemotherapy, as these lymphomas do not always require intervention and can be managed with “watchful waiting” in asymptomatic cases [26]. Given the rarity of these cases, with only a few hundred reported in the literature [27], our study contributes further insights into this interesting subgroup of GI lymphomas.

The clinical imperative of our study lies in identifying diagnostic “red flags” amidst nonspecific symptoms. While symptoms mimic other GI entities, we found that diarrhea and weight loss are significantly more prevalent in T-cell DL, paralleling the profile of EATL. Endoscopically, although villous shortening remains a key indicator for T-cell subtypes, our data suggest a paradigm shift: no single feature is diagnostic, and the high degree of overlapping morphologies mandates a “low threshold for biopsy” approach for any duodenal mucosal abnormality. Gastrointestinal lymphomas may mimic several other clinicopathological entities of the gastrointestinal tract [28]. Recognizing individuals at risk is a clinical imperative to provide a definitive diagnosis and commence appropriate therapy, but achieving this in practice is often challenging. Mirroring symptoms reported for gastrointestinal lymphomas in general [28], clinical presentations for DLs were generally nonspecific. Few clinical signals might differentiate different types of DLs, although diarrhea and weight loss are both significantly more common presenting symptoms in patients with T-cell lymphoma, consistent with the clinical symptom profile for EATL, which includes diarrhea in 30–50% of cases and weight loss in 30–80% of cases [22]. Similarly, DLs present with a range of endoscopic features. Although the only endoscopic feature associated with the type of lymphoma was villous shortening, which was present in 57.1% of T-cell lymphomas but only 8.3% of B-cell lymphomas, the spectrum of endoscopic features observed here is consistent with other reports of the endoscopic features associated with DL. In their comprehensive review, Iwamuro et al. [14] described typical endoscopic features of different subtypes of DL, wherein follicular lymphoma was characterized by multiple white nodular submucosal granules [29]; DLBCL with ulcerating, protruding lesions [30]; MALT lymphoma with nodular, ulcerating, flat depressed, or subepithelial lesions, together with similar white nodularity that must be distinguished from follicular lymphoma [31]; and mantle-cell lymphoma with lymphomatous polyposis or protruding lesions [14]. A more general survey of 140 lymphomas throughout the gastrointestinal tract, only five of which were in the duodenum, revealed similar endoscopic heterogeneity, with superficial and protruding/ulcerating forms also commonly observed [32]. In that series, fungating and protruding/ulcerating lesions were more frequently associated with aggressive lymphomas such as DLBCL, MCL, and T-cell lymphoma. Our data suggest that, for duodenal lesions, there are no defining endoscopic features and that their subtypes share overlapping features. All suspicious lesions in the duodenum must be biopsied.

Data on prognostic indicators in DLs are limited. Our multivariable model provides a critical comparative insight: while the SEER analysis identified standard factors such as age and stage, our study introduces “granularity” as a novel endoscopic prognosticator (HR 0.33). This pattern was independent of histopathological subtype, suggesting that macroscopic mucosal changes may reflect the underlying tissue architecture and tumor–host interface. From a practical standpoint, the presence of granularity could serve as a visual cue for a more indolent disease course or a higher susceptibility to chemotherapy, potentially aiding clinicians in risk-stratifying patients at the time of initial endoscopy. Furthermore, our study confirms that despite the aggressive nature of DL, chemotherapy remains a cornerstone of management, significantly reducing the risk of death (HR: 0.22) across all stages. The SEER analysis identified age, sex, Ann Arbor stage, and histological type as independent prognostic factors for disease-specific survival (all *p* < 0.05). Our multivariable model suggested that a granular appearance at endoscopy and receiving chemotherapy were associated with a reduced risk of death from DLs, whereas T-cell lymphoma and stage IV lymphoma were both associated with an increased risk of death from the disease. Our findings, therefore, confirm the poor prognostic outlook for T-cell lymphomas and high-grade disease, as expected, and that, similar to other primary intestinal lymphomas, chemotherapy has a role in the management of DLs [33]. The new finding that a granular appearance at endoscopy was a favorable prognostic factor in both univariable and multivariable analyses is interesting. This pattern, which most closely resembled the diffuse and focal mucosal granularity observed in some adult T-cell leukemia/lymphoma lymphomas [34] (Figure 1G), was distinct from the white nodular granularity described for follicular lymphoma [29] and was independent of and observed in every histopathological subtype. The significance of this observation is uncertain, but it may provide a macroscopic representation of the lesion’s tissue distribution associated with a more indolent course or susceptibility to therapy. Furthermore, independent validation of this observation is needed, and future studies should specifically document this endoscopic pattern.

Despite providing one of the most comprehensive integrated analyses of DL, this study had several inherent limitations that warrant critical reflection. First, regarding methodological constraints, as a retrospective observational study spanning two decades, potential information bias and inconsistencies in standardized reporting were inevitable. While we conducted a rigorous expert re-evaluation of all images, the technical evolution of endoscopic and histopathological diagnostic criteria since 2002 may have introduced historical bias. Second, dataset-related issues, particularly the relatively small sample size (*N* = 50), limited our ability to perform robust subgroup analyses or complex cross-validation for the survival models. Additionally, our cohort exhibited a notable selection bias, as our institution serves as a national tertiary referral hub for rare and complex cases, leading to a high prevalence of aggressive T-cell subtypes (28%) that may not accurately reflect the disease distribution in primary care settings or other ethnicities. While these results are highly relevant for Asian cohorts, they should be applied with caution in Western populations where B-cell lymphomas are predominant, as the disease distribution differs significantly across ethnicities.

The primary challenges encountered during this research involved the retrospective harmonization of multimodal data and the identification of long-term follow-up details for this transient patient population. Future research should aim for multi-center prospective collaborations to validate granularity as a universal prognostic biomarker and to expand the cohort size for more powerful machine learning-based risk stratification. Furthermore, integrating genomic and proteomic data with endoscopic phenotypes could further elucidate the biological mechanisms driving the diverse outcomes observed in duodenal lymphomas.

## 5. Conclusions

In conclusion, this study represents the first integrated clinicopathological and endoscopic analysis of DL within a large-scale, 20-year cohort in China. By meticulously harmonizing long-term follow-up data with expert-led diagnostic re-evaluations, we identified unique disease patterns specifically relevant to the Asian population, notably the high prevalence and dismal prognosis of T-cell subtypes such as MEITL. Our findings establish that while DL presents with heterogeneous and nonspecific endoscopic features, the identification of “mucosal granularity” serves as a robust, independent indicator of favorable overall survival.

These results possess significant clinical implications, suggesting that detailed endoscopic characterization can provide immediate, actionable insights for patient risk stratification at the time of initial diagnosis. Furthermore, our analysis confirms the critical role of chemotherapy in improving survival outcomes across diverse DL subtypes. By providing a structured framework for the diagnosis and prognostic assessment of this rare malignancy, this work contributes to closing the existing knowledge gap and underscores the necessity of a low threshold for biopsy in suspicious duodenal lesions to ensure timely and precise intervention.

## Figures and Tables

**Table 1:** Clinicopathological characteristics of the study population. The values are *n* (%) unless otherwise stated.

Characteristic	*N* = 50
Sex	
Female	22 (44%)
Male	28 (56%)
Age, years (IQR)	56 (46, 70)
Lymphoma type	
B-cell, indolent	16 (32%)
B-cell, aggressive	20 (40%)
T-cell	14 (28%)
Histopathological subtype	
DLBCL	18 (36%)
Mantle-cell lymphoma	2 (4.0%)
MALT	8 (16%)
Follicular lymphoma	8 (16%)
Peripheral T-cell lymphoma	1 (2.0%)
T-cell lymphoblastic lymphoma	1 (2.0%)
Enteropathy-type T-cell lymphoma	4 (8.0%)
T-cell unclassified	8 (16%)
Ann Arbor stage simplified	
II	10 (20%)
III	5 (10%)
IV	35 (70%)
Clinical presentation	
*Helicobacter pylori* (*H. pylori)* status	
*H. pylori*-negative	35 (70%)
*H. pylori*-positive	15 (30%)
Fever	11 (22%)
Abdominal distension	12 (24%)
Abdominal pain	25 (50%)
Diarrhea	15 (30%)
Difficulty eating	1 (2.0%)
Abdominal mass	1 (2.0%)
Poor appetite	9 (18%)
Vomiting	12 (24%)
Rectal bleeding	
No hematochezia	41 (82%)
Melena	6 (12%)
Hematochezia	3 (6.0%)
Difficulty defecating	3 (6.0%)
>5 kg weight loss	33 (66%)
Chemotherapy	31 (62%)
Surgery	3 (6.0%)
Survival	
Died	32 (64%)
Alive	18 (36%)
Follow-up, months (IQR)	20 (3, 38)
Survival, months (IQR)	24.1 (13, 117)

**Table 2:** Endoscopic features of patients with duodenal lymphoma.

Characteristic	*N* = 50
Lesion location	
Duodenal bulb	9 (18%)
Descending duodenum	17 (34%)
Descending duodenum and bulb	19 (38%)
Horizontal duodenum	3 (6.0%)
Descending and horizontal duodenum	2 (4.0%)
Other sites of gastrointestinal involvement	
Esophageal involvement	1 (2.0%)
Stomach involvement	11 (22%)
Duodenal involvement alone	36 (72%)
Jejunum/ileum involvement	2 (4.0%)
End of ileum involvement	5 (10%)
Granularity	24 (48%)
Villous shortening	11 (22%)
Shallow folds	12 (24%)
Infiltrative change	13 (26%)
Polyp-like protrusion	5 (10%)
Mass-forming	20 (40%)
Lymphangiectasia	16 (32%)
Mucosal erosion	12 (24%)
Ulceration	
No ulceration	29 (58%)
Superficial ulceration	16 (32%)
Deep ulceration	5 (10%)
Ulcer number	
No ulceration	29 (58%)
Single ulceration	7 (14%)
Multiple ulcerations	14 (28%)

**Table 3:** Multivariate Cox regression analysis of predictors of OS in patients with primary duodenal lymphoma.

Variable		HR (Univariable)	HR (Multivariable)
Sex	Female	-	-
	Male	1.81 (0.87–3.77, *p* = 0.111)	1.42 (0.55–3.71, *p* = 0.470)
Weight loss	No weight loss	-	-
	>5 kg weight loss	2.02 (0.93–4.41, *p* = 0.076)	0.71 (0.20–2.48, *p* = 0.591)
Lesion location	Duodenal bulb	-	-
	Descending duodenum	0.54 (0.20–1.47, *p* = 0.229)	0.94 (0.22–3.92, *p* = 0.930)
	Horizontal duodenum	0.89 (0.18–4.33, *p* = 0.887)	0.72 (0.10–5.34, *p* = 0.748)
	Descending duodenum and bulb	0.75 (0.30–1.89, *p* = 0.543)	0.56 (0.14–2.31, *p* = 0.425)
	Descending and horizontal duodenum	0.45 (0.06–3.67, *p* = 0.456)	2.01 (0.15–27.44, *p* = 0.599)
Granularity	No granularity	-	-
	Granularity	0.45 (0.22–0.92, *p* = 0.029)	0.33 (0.11–0.99, *p* = 0.049)
Lymphangiectasia	No lymphangiectasia	-	-
	Lymphangiectasia	0.61 (0.28–1.37, *p* = 0.235)	0.53 (0.18–1.60, *p* = 0.261)
Ulceration present/absent	No ulceration	-	-
	Ulceration	1.59 (0.79–3.22, *p* = 0.197)	0.55 (0.19–1.59, *p* = 0.269)
Chemotherapy	No	-	-
	Yes	0.68 (0.33–1.38, *p* = 0.282)	0.22 (0.07–0.69, *p* = 0.010)
Surgery	No surgery	-	-
	Underwent surgery	2.75 (0.81–9.36, *p* = 0.105)	0.94 (0.12–7.27, *p* = 0.954)
Lymphoma type	Aggressive B-cell	-	-
	Indolent B-cell	0.23 (0.07–0.68, *p* = 0.008)	0.35 (0.07–1.71, *p* = 0.195)
	T-cell	2.27 (1.05–4.90, *p* = 0.037)	9.19 (2.12–39.83, *p* = 0.003)
Ann Arbor stage simplified	II	-	-
	III	2.16 (0.44–10.71, *p* = 0.346)	3.88 (0.55–27.32, *p* = 0.173)
	IV	3.49 (1.05–11.57, *p* = 0.041)	12.76 (1.70–95.66, *p* = 0.013)

## Data Availability

The data presented in this study are available on request from the corresponding author. The data are not publicly available due to ethical reasons.

## Supplementary Materials

The following supporting information can be downloaded at https://www.mdpi.com/article/10.3390/diagnostics16081173/s1: Figure S1: Survival outcome in patients with different T cell lymphomas; Figure S2: Survival outcome in patients with follicular lymphoma (blue) vs MALToma (red) (A) and follicular lymphoma (blue) vs other B cell lymphomas (red) (B); Figure S3: Survival outcome in patients with T cell lymphomas (A), aggressive B cell lymphomas (B), and indolent B cell lymphomas (C) with chemotherapy (red lines) and without chemotherapy (blue lines); Table S1: Image assessment of endoscopic features in 5 typical cases by the 2 senoir endoscopists; Table S2: Association between administration of chemotherapy and Ann Arbor stage; Table S3: Univariable associations between clinical parameters and OS in 50 patients with primary duodenal lymphoma; Table S4: Univariable associations between endoscopic parameters and OS in 50 patients with primary duodenal lymphoma; Table S5: Univariable associations between pathology parameters and OS in 50 patients with primary duodenal lymphoma.

## Abbreviations

The following abbreviations were used in this manuscript:

CE	Chromoendoscopy
DLBCL	Diffuse Large B-Cell Lymphoma
DLs	Duodenal Lymphomas
EATLs	Enteropathy-Associated T-Cell Lymphomas
GDPL	Gemcitabine, Dexamethasone, Cisplatin, and L-Asparaginase
GI	Gastrointestinal
IQRs	Interquartile Ranges
MALT	Mucosa-Associated Lymphoid Tissue
MCL	Mantle-Cell Lymphoma
MEITL	Monomorphic Epitheliotropic Intestinal T-Cell Lymphoma
ME-NBI	Magnifying Endoscopy with Narrow-Band Imaging
NHL	Non-Hodgkin’s Lymphoma
OS	Overall Survival
R-CHOP	Rituximab, Cyclophosphamide, Doxorubicin, Vincristine, and Prednisone
SEER	Surveillance, Epidemiology, and End Results
WHO	The World Health Organization
WLE	White-Light Endoscopy

## References

[1] Nakamura S., Matsumoto T. (2013). Gastrointestinal lymphoma: Recent advances in diagnosis and treatment. Digestion.

[2] Andrews C.N., Gill M.J., Urbanski S.J., Stewart D., Perini R., Beck P. (2008). Changing epidemiology and risk factors for gastrointestinal non-Hodgkin’s lymphoma in a North American population: Population-based study. Am. J. Gastroenterol..

[3] Howell J., Auer-Grzesiak I., Zhang J., Andrews C., Stewart D., Urbanski S. (2012). Increasing incidence rates, distribution and histological characteristics of primary gastrointestinal non-Hodgkin lymphoma in a North American population. Can. J. Gastroenterol. Hepatol..

[4] Ding W., Zhao S., Wang J., Yang Q., Sun H., Yan J., Gao L., Yao W., Zhang W., Liu W. (2016). Gastrointestinal Lymphoma in Southwest China: Subtype Distribution of 1,010 Cases Using the WHO (2008) Classification in a Single Institution. Acta Haematol..

[5] Nakamura S., Matsumoto T., Iida M., Yao T., Tsuneyoshi M. (2003). Primary gastrointestinal lymphoma in Japan: A clinicopathologic analysis of 455 patients with special reference to its time trends. Cancer.

[6] Cho S.J., Ryu K.W., Kim C.G., Lee J.Y., Kook M.C., Min H.S., Choi I.J. (2008). Duodenal mucosa-associated lymphoid tissue lymphoma masquerading as an ulcer scar. Endoscopy.

[7] Iwamuro M., Kondo E., Otsuka F., Takata K., Yoshino T., Kawahara Y., Okada H. (2016). Detection of Minute Duodenal Follicular Lymphoma Lesions Using Magnifying Endoscopy. Acta Medica Okayama.

[8] Tari A., Sato Y., Asaoku H., Kunihiro M., Fukumoto A., Tanaka S., Fujihara M., Yoshino T. (2010). A duodenal follicular lymphoma associated with the lesion mimicking MALT lymphoma in terminal ileum and Bauhin valve. Med. Mol. Morphol..

[9] Trivedi P., Gupta A., Pasricha S. (2012). Primary diffuse large B-cell lymphoma of ampulla of vater: A rare case report. J. Gastrointest. Cancer.

[10] Zheng Q.F., Li J.Y., Qin L., Wei H.M., Cai L.Y., Nong B. (2018). Gastrointestinal involvement by mantle cell lymphoma identified by biopsy performed during endoscopy: A case report. Medicine.

[11] Zheng G., Wang Y., Zhao Y., Zheng Z. (2020). Clinicopathological Features, Treatment Strategy, and Prognosis of Primary Non-Hodgkin’s Lymphoma of the Duodenum: A SEER Database Analysis. Can. J. Gastroenterol. Hepatol..

[12] Karanam P.K., Al-Hamadani M., Go R.S. (2016). Enteropathy-associated T-cell lymphoma in the US: Higher incidence and poorer survival among Asians. Br. J. Haematol..

[13] Chan J.K., Chan A.C., Cheuk W., Wan S.K., Lee W.K., Lui Y.H., Chan W.K. (2011). Type II enteropathy-associated T-cell lymphoma: A distinct aggressive lymphoma with frequent gammadelta T-cell receptor expression. Am. J. Surg. Pathol..

[14] Iwamuro M., Tanaka T., Okada H. (2023). Review of lymphoma in the duodenum: An update of diagnosis and management. World J. Gastroenterol..

[15] Müller A.M., Ihorst G., Mertelsmann R., Engelhardt M. (2005). Epidemiology of non-Hodgkin’s lymphoma (NHL): Trends, geographic distribution, and etiology. Ann. Hematol..

[16] von Elm E., Altman D.G., Egger M., Pocock S.J., Gøtzsche P.C., Vandenbroucke J.P., STROBE Initiative (2014). The Strengthening the Reporting of Observational Studies in Epidemiology (STROBE) Statement: Guidelines for reporting observational studies. Int. J. Surg..

[17] Alaggio R., Amador C., Anagnostopoulos I., Attygalle A.D., Araujo I.B.d.O., Berti E., Bhagat G., Borges A.M., Boyer D., Calaminici M. (2022). The 5th edition of the World Health Organization Classification of Haematolymphoid Tumours: Lymphoid Neoplasms. Leukemia.

[18] Bendel R.B., Afifi A.A. (1977). Comparison of stopping rules in forward “stepwise” regression. J. Am. Stat. Assoc..

[19] Mickey R.M., Greenland S. (1989). The impact of confounder selection criteria on effect estimation. Am. J. Epidemiol..

[20] Lagoo A.S., Cardona D.M., Layne A. (2012). Lymphomas of the gastro-intestinal tract—Pathophysiology, pathology, and differential diagnosis. Indian J. Pathol. Microbiol..

[21] Fujishima F., Katsushima H., Fukuhara N., Konosu-Fukaya S., Nakamura Y., Sasano H., Ichinohasama R. (2018). Incidence Rate, Subtype Frequency, and Occurrence Site of Malignant Lymphoma in the Gastrointestinal Tract: Population-Based Analysis in Miyagi, Japan. Tohoku J. Exp. Med..

[22] Ondrejka S., Jagadeesh D. (2016). Enteropathy-Associated T-Cell Lymphoma. Curr. Hematol. Malig. Rep..

[23] Tse E., Gill H., Loong F., Kim S.J., Ng S., Tang T., Ko Y., Chng W., Lim S., Kim W.S. (2012). Type II enteropathy-associated T-cell lymphoma: A multicenter analysis from the Asia Lymphoma Study Group. Am. J. Hematol..

[24] Ferreri A.J., Zinzani P.L., Govi S., Pileri S.A. (2011). Enteropathy-associated T-cell lymphoma. Crit. Rev. Oncol. Hematol..

[25] Veloza L., Cavalieri D., Missiaglia E., Ledoux-Pilon A., Bisig B., Pereira B., Bonnet C., Poullot E., Quintanilla-Martinez L., Dubois R. (2023). Monomorphic epitheliotropic intestinal T-cell lymphoma comprises morphologic and genomic heterogeneity impacting outcome. Haematologica.

[26] Small S., Barnea Slonim L., Williams C., Karmali R. (2021). B Cell Lymphomas of the GI Tract. Curr. Gastroenterol. Rep..

[27] Takata K., Miyata-Takata T., Sato Y., Iwamuro M., Okada H., Tari A., Yoshino T. (2018). Gastrointestinal follicular lymphoma: Current knowledge and future challenges. Pathol. Int..

[28] Thomas A.S., Schwartz M., Quigley E. (2019). Gastrointestinal lymphoma: The new mimic. BMJ Open Gastroenterol..

[29] Iwamuro M., Okada H., Takata K., Nose S., Miyatani K., Yoshino T., Yamamoto K. (2014). Diagnostic accuracy of endoscopic biopsies for the diagnosis of gastrointestinal follicular lymphoma: A clinicopathologic study of 48 patients. Ann. Diagn. Pathol..

[30] Vetro C., Romano A., Amico I., Conticello C., Motta G., Figuera A., Chiarenza A., Di Raimondo C., Giulietti G., Bonanno G. (2014). Endoscopic features of gastro-intestinal lymphomas: From diagnosis to follow-up. World J. Gastroenterol..

[31] Na H.K., Won S.H., Ahn J.Y., Kim G.H., Jung K.W., Lee J.H., Kim D.H., Choi K.D., Song H.J., Lee G.H. (2021). Clinical course of duodenal mucosa-associated lymphoid tissue lymphoma: Comparison with gastric mucosa-associated lymphoid tissue lymphoma. J. Gastroenterol. Hepatol..

[32] Tran Q.T., Nguyen Duy T., Nguyen-Tran B.S., Nguyen-Thanh T., Ngo Q.T., Tran Thi N.P., Le V., Dang-Cong T. (2023). Endoscopic and Histopathological Characteristics of Gastrointestinal Lymphoma: A Multicentric Study. Diagnostics.

[33] Ghimire P., Wu G.Y., Zhu L. (2011). Primary gastrointestinal lymphoma. World J. Gastroenterol..

[34] Miike T., Kawakami H., Kameda T., Yamamoto S., Tahara Y., Hidaka T., Kubuki Y., Yorita K., Akiyama Y., Arimura Y. (2020). Clinical characteristics of adult T-cell leukemia/lymphoma infiltration in the gastrointestinal tract. BMC Gastroenterol..

