# Supplementary material for: Duodenal Lymphomas: Comprehensive Evaluation of Endoscopic Features and Clinical Outcomes in a Tertiary Center
Source: Diagnostics (Basel). 2026 Apr 15;16(8):1173. doi: 10.3390/diagnostics16081173 (PMC13114455; doi:10.3390/diagnostics16081173)

SUPPLEMENTARY INFORMATION

Supplementary Table S1. Image assessment of endoscopic features in 5 typical cases by the 2 senoir endoscopists.

**Endoscopic features**	**Endoscopist 1**					**Endoscopist 2**				
	**Case 1**	**Case 2**	**Case 3**	**Case 4**	**Case 5**	**Case 1**	**Case 2**	**Case 3**	**Case 4**	**Case 5**
Granularity	1	1	0	1	1	1	1	0	1	1
Villous shortening	1	0	0	1	0	1	1	0	0	0
Shallow folds	1	0	0	0	0	1	0	0	0	0
Infiltrative change	0	0	0	0	1	0	0	0	0	1
Polyp-like protrusion	0	1	0	0	0	0	1	0	0	0
Mass-forming	0	0	1	1	0	0	0	1	1	0
Lymphangiectasia	0	0	1	0	0	0	0	1	0	0
Ulceration	0	0	1	1	1	0	0	1	1	1

Supplementary Table S2. Association between administration of chemotherapy and Ann Arbor stage.

		**Ann Arbor Stage**			
**Chemotherapy**		**II**	**III**	**IV**	**Total**
No	Number	6	4	9	19
	%	31.6%	21.1%	47.4%	100.0%
Yes	Number	4	1	26	31
	%	12.9%	3.2%	83.9%	100.0%
Total	Number	10	5	35	50
	%	20.0%	10.0%	70.0%	100.0%

Supplementary Table S3. Univariable associations between clinical parameters and OS in 50 patients with primary duodenal lymphoma.

**Variable**			**HR (univariable)**
Sex	Female	22 (44.0)	-
	Male	28 (56.0)	1.81 (0.87-3.77, p=0.111)
H. pylori status	*H. pylori* negative	35 (70.0)	-
	H. pylori positive	15 (30.0)	1.30 (0.63-2.71, p=0.478)
Fever	No fever	39 (78.0)	-
	Fever	11 (22.0)	0.91 (0.39-2.11, p=0.829)
Abdominal distension	No abdominal distension	38 (76.0)	-
	Abdominal distension	12 (24.0)	1.05 (0.45-2.42, p=0.915)
Abdominal pain	No abdominal pain	25 (50.0)	-
	Abdominal pain	25 (50.0)	1.46 (0.72-2.95, p=0.289)
Diarrhea	No diarrhea	35 (70.0)	-
	Diarrhea	15 (30.0)	1.43 (0.69-2.97, p=0.341)
Difficulty eating	No difficulty eating	49 (98.0)	-
	Difficulty eating	1 (2.0)	0.00 (0.00-Inf, p=0.997)
Abdominal mass	No abdominal mass	49 (98.0)	-
	Abdominal mass	1 (2.0)	0.00 (0.00-Inf, p=0.997)
Appetite	Normal appetite	41 (82.0)	-
	Poor appetite	9 (18.0)	1.32 (0.54-3.24, p=0.547)
Vomiting	No vomiting	38 (76.0)	-
	Vomiting	12 (24.0)	0.88 (0.39-1.97, p=0.756)
Rectal bleeding	No hematochezia	41 (82.0)	-
	Melena	6 (12.0)	1.44 (0.55-3.79, p=0.455)
	Hematochezia	3 (6.0)	1.11 (0.26-4.69, p=0.891)
Defecation	No difficulty defecating	47 (94.0)	-
	Difficulty defecating	3 (6.0)	0.40 (0.05-2.92, p=0.366)
Weight loss	No weight loss	17 (34.0)	-
	>5 kg weight loss	33 (66.0)	2.02 (0.93-4.41, p=0.076)
Age	Mean (SD)	56.2 (15.5)	0.99 (0.97-1.01, p=0.472)

Supplementary Table S4. Univariable associations between endoscopic parameters and OS in 50 patients with primary duodenal lymphoma.

Variable			HR (univariable)
Lesion location	Duodenal bulb	9 (18.0)	-
	Descending duodenum	17 (34.0)	0.54 (0.20-1.47, p=0.229)
	Horizontal duodenum	3 (6.0)	0.89 (0.18-4.33, p=0.887)
	Descending duodenum and bulb	19 (38.0)	0.75 (0.30-1.89, p=0.543)
	Descending and horizontal duodenum	2 (4.0)	0.45 (0.06-3.67, p=0.456)
Granularity	No granular appearance	26 (52.0)	-
	With granular appearance	24 (48.0)	0.45 (0.22-0.92, p=0.029)
Villous shortening	No villous shortening	39 (78.0)	-
	Villous shortening	11 (22.0)	1.23 (0.55-2.74, p=0.615)
Shallow folds	No shallow folds	38 (76.0)	-
	Shallow folds	12 (24.0)	0.73 (0.32-1.70, p=0.471)
Infiltrative change	No infiltrative change	37 (74.0)	-
	Infiltrative change	13 (26.0)	1.23 (0.55-2.76, p=0.611)
Polyp-like protrusion	No polyp-like protrusion	45 (90.0)	-
	Polyp-like protrusion	5 (10.0)	0.84 (0.25-2.77, p=0.768)
Mass-forming	No mass-forming changes	30 (60.0)	-
	Mass-forming changes	20 (40.0)	1.16 (0.58-2.35, p=0.673)
Lymphangiectasia	No lymphangiectasia	34 (68.0)	-
	Lymphangiectasia	16 (32.0)	0.61 (0.28-1.37, p=0.235)
Mucosal erosion	No mucosal erosion	38 (76.0)	-
	Mucosal erosion	12 (24.0)	1.41 (0.62-3.20, p=0.413)
Ulceration present/absent	No ulceration	29 (58.0)	-
	Ulceration	21 (42.0)	1.59 (0.79-3.22, p=0.197)
Ulceration	No ulceration	29 (58.0)	-
	Superficial ulceration	16 (32.0)	1.45 (0.68-3.12, p=0.340)
	Deep ulceration	5 (10.0)	2.23 (0.73-6.84, p=0.161)
Ulcer number	No ulceration	29 (58.0)	-
	Single ulceration	7 (14.0)	0.67 (0.20-2.29, p=0.521)
	Multiple ulceration	14 (28.0)	2.60 (1.18-5.71, p=0.018)
End of ileum involvement	No end of ileum involvement	45 (90.0)	-
	End of ileum involvement	5 (10.0)	0.88 (0.27-2.89, p=0.831)
Other sites of involvement	Esophageal involvement	1 (2.0)	-
	Stomach involvement	11 (22.0)	0.92 (0.11-7.70, p=0.936)
	Duodenal involvement alone	36 (72.0)	1.13 (0.15-8.39, p=0.907)
	Jejunum/ileum involvement	2 (4.0)	0.77 (0.05-12.28, p=0.851)

Supplementary Table S5. Univariable associations between pathology parameters and OS in 50 patients with primary duodenal lymphoma.

**Variable**			**HR (univariable)**
Chemotherapy	No	19 (38.0)	-
	Yes	31 (62.0)	0.68 (0.33-1.38, p=0.282)
Surgery	No surgery	47 (94.0)	-
	Underwent surgery	3 (6.0)	2.75 (0.81-9.36, p=0.105)
Lymphoma type	Aggressive B cell	20 (40.0)	-
	Indolent B cell	16 (32.0)	0.23 (0.07-0.68, p=0.008)
	T cell	14 (28.0)	2.27 (1.05-4.90, p=0.037)
Histopathological subtype	DLBCL	18 (36.0)	-
	Follicular lymphoma	8 (16.0)	0.12 (0.02-0.88, p=0.038)
	MALT	8 (16.0)	0.35 (0.10-1.22, p=0.099)
	Marginal cell lymphoma	2 (4.0)	1.38 (0.31-6.20, p=0.673)
	Peripheral T cell lymphoma	1 (2.0)	1.10 (0.14-8.54, p=0.926)
	T cell lymphoblastic lymphoma	1 (2.0)	3.84 (0.47-31.76, p=0.212)
	Enteropathy-type T cell lymphoma	4 (8.0)	1.24 (0.35-4.37, p=0.741)
	T cell unclassified	8 (16.0)	5.09 (1.89-13.74, p=0.001)
Ann Arbor Stage Simplified	II	10 (20.0)	-
	III	5 (10.0)	2.16 (0.44-10.71, p=0.346)
	IV	35 (70.0)	3.49 (1.05-11.57, p=0.041)
Ki67 index	2	10 (20.0)	-
	3	5 (10.0)	2.16 (0.44-10.71, p=0.346)
	4	35 (70.0)	3.49 (1.05-11.57, p=0.041)

Supplementary Figure S1. Survival outcome in patients with different T cell lymphomas.

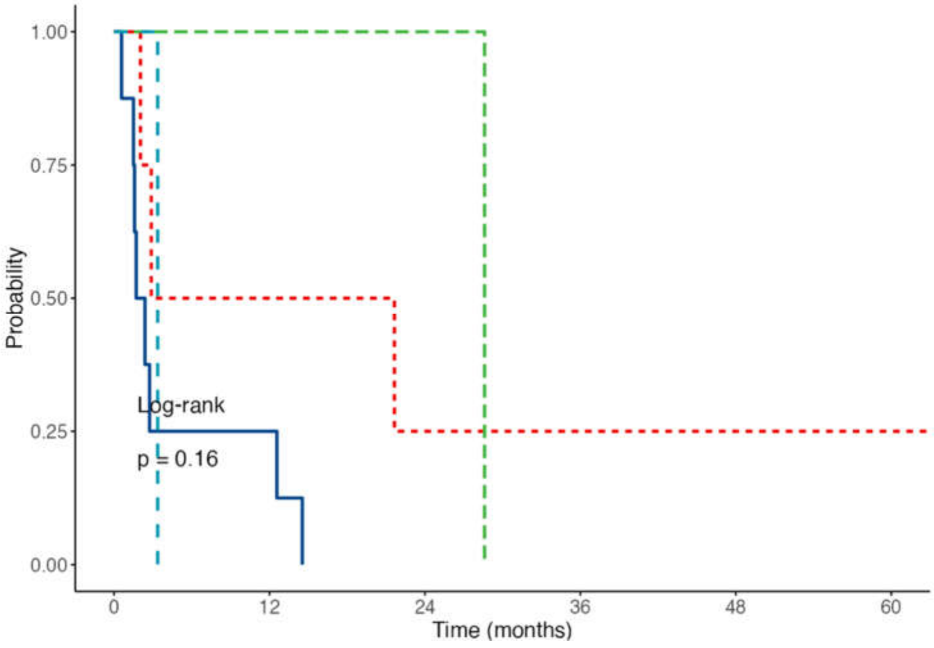

Supplementary Figure S2. Survival outcome in patients with follicular lymphoma (blue) vs MALToma (red) (A) and follicular lymphoma (blue) vs other B cell lymphomas (red) (B).

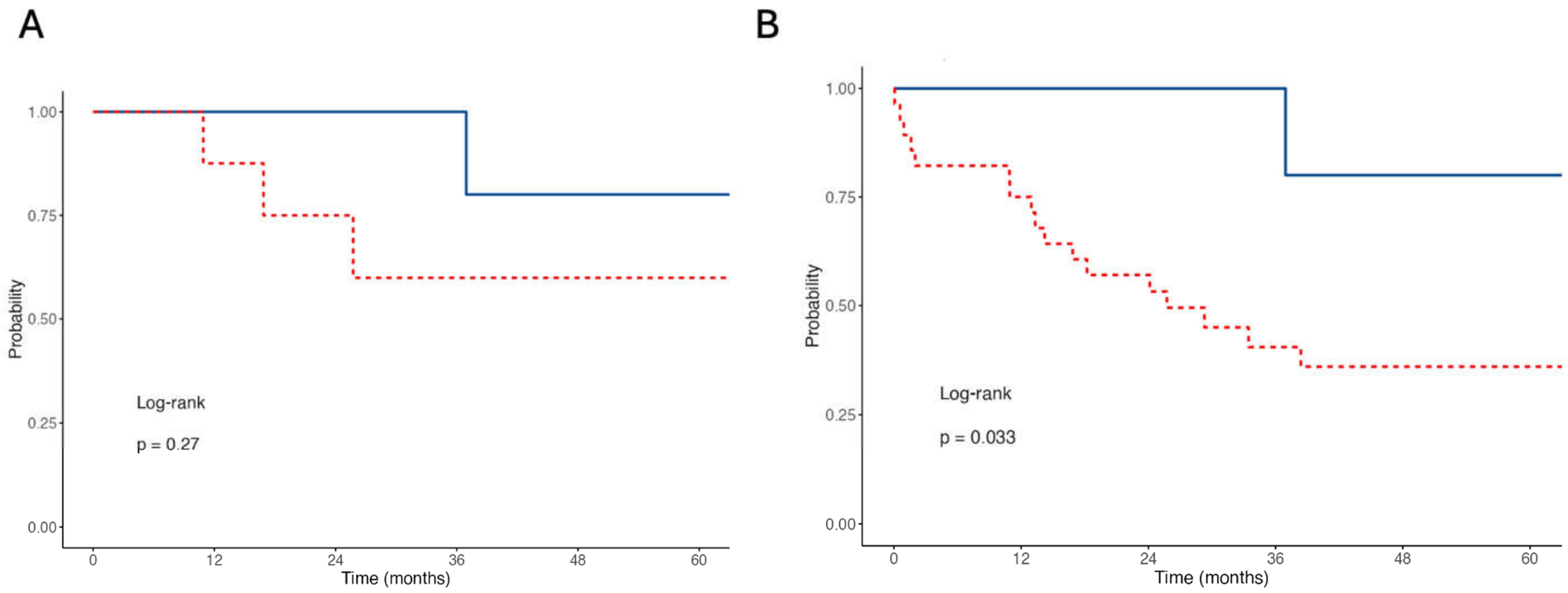

Supplementary Figure S3. Survival outcome in patients with T cell lymphomas (A), aggressive B cell lymphomas (B), and indolent B cell lymphomas (C) with chemotherapy (red lines) and without chemotherapy (blue lines).

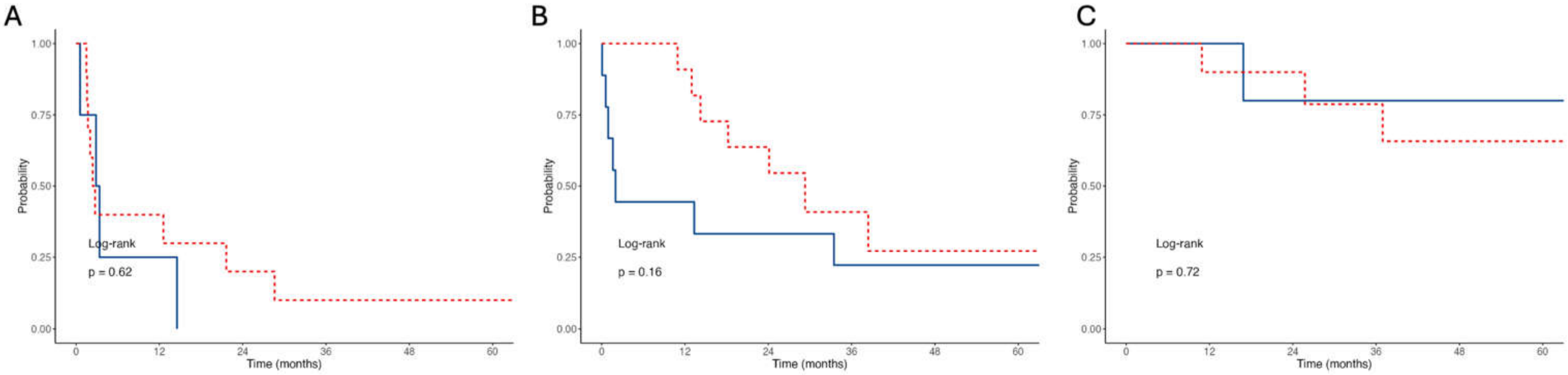

Supplement: Supplementary file 1 [file diagnostics-16-01173-s001.zip › diagnostics-4142264-supplementary.pdf]
